# Inoculum Concentration and Mineral Fertilization: Effects on the Endophytic Microbiome of Soybean

**DOI:** 10.3389/fmicb.2022.900980

**Published:** 2022-07-07

**Authors:** Roberta Mendes dos Santos, Luis Gabriel Cueva-Yesquén, Fabiana Fantinatti Garboggini, Nicolas Desoignies, Everlon Cid Rigobelo

**Affiliations:** ^1^Graduate Program in Agricultural Microbiology, Faculty of Agrarian and Veterinary Sciences, State University of Sao Paulo (UNESP), Jaboticabal, Brazil; ^2^Agricultural and Livestock Microbiology Graduation Program, São Paulo State University (UNESP), School of Agricultural and Veterinarian Sciences, Jaboticabal, São Paulo, Brazil; ^3^Phytopathology, Microbial and Molecular Farming Lab, Centre d’Etudes et de Recherche Appliquée -Haute Ecole Provinciale du Hainaut Condorcet, Ath, Belgium

**Keywords:** soybean, root microbiome, microbial diversity, inoculant, *Bacillus subtilis*

## Abstract

Soybean crops are of great economic importance worldwide and in Brazil. This crop is a commodity that provides large amounts of financial resources to the country. Soybean productivity is influenced by several biotic and abiotic factors, and most of these factors cannot be controlled by agricultural practices. Due to the soybean cultivars used and their required yields, the soybean crop, similar to other agriculturally important crops, requires large amounts of mineral fertilizers. There are several microorganisms that colonize soybean plant roots without causing symptoms or damage. These microorganisms that colonize plant tissues are called endophytes and can often promote plant growth and development. Little is known about the factors that influence endophyticism. The aim of the present study was to evaluate whether *Bacillus subtilis* inoculant concentrations and levels of mineral fertilization recommended for the crop have any influence on the endophytic microbiome of soybean plant roots. The results show that *B. subtilis* inoculations did not affect the endophytic community of the roots; however, the evaluation of the microbial community structure according to the alpha diversity metrics observed richness, Chao1 index, Shannon index and Simpson index showed that microbial diversity of endophytes was higher at fertilization levels of 50 and 100%, with a significant difference (*p* < 0.05) between 0 and 50% and 0 and 100% fertilization.

## Introduction

Microorganisms have been associated with plants since their early evolution; these microorganisms can have different types of interactions that can be beneficial, harmful or neutral (Del Carmen Orozco-Mosqueda and Santoyo, 2021; Roberts, 2022). When the relationship is harmful to the plant, it results in a decrease in plant development, while a beneficial relationship promotes development (Husseiny et al., 2021; Tao et al., 2022). Bacteria that interact with plants are classified according to their location and can be rhizospheric, epiphytic and endophytic. Endophytic bacteria have the ability to invade the internal tissues of living plants without producing disease symptoms during part of all of their life cycle (Adeleke et al., 2021).

The endophytic environment is more protected than the soil or rhizospheric environment and provides an ecological advantage to rhizospheric colonizing bacteria (Guzmán-Guzmán and Santoyo, 2022). However, in plant endophyte interactions, bacteria do not reside within cells and do not induce the formation of differentiated plant structures, such as nodules, as in legume-rhizobium interactions (Brader et al., 2017; Lyu et al., 2021). Bacteria within the host find high levels of nutrients, low competition and protection against environmental stress, and in some cases, living endophytically can guarantee their dispersion by vertical transfer (Compant et al., 2021). The life of bacteria within plant tissues allows for a more intimate interaction with the host, effectively influencing the plant phenotype (Compant et al., 2021).

There is strong evidence that many apparently commensalistic endophytes can also promote plant growth and defense (Santoyo et al., 2016; Hossain et al., 2017), but the ecology and functions of these beneficial endophytes are not well understood. In particular, endophytic plant growth-promoting bacteria (PGP) can promote plant growth by mechanisms that include the release of phytohormones (Chouhan et al., 2021), nitrogen fixation (Sun et al., 2022), improved mineral acquisition (Kushwaha et al., 2022), the production of growth-promoting compounds (Kumar et al., 2019) and increased stress tolerance (Aziz et al., 2022).

The endophytic bacterium *Bacillus subtilis* has been used to improve soybean production due to its many attributes related to plant growth. These attributes have been used for the biological control of seed pathogens and to promote plant growth (Araujo et al., 2005), modulate the root architecture, and improve the absorption of water and nutrients from the soil (Bavaresco et al., 2020), and to enhance the physiological parameters triggering various defense responsive enzymes. Escobar Diaz et al. (2021b) studied different inoculant concentrations in cotton crops and verified that the high inoculant concentrations of different isolates of *B. subtilis* and two isolates of *Aspergillus* sp. did not affect the number of colony-forming units (CFUs) or affect plant growth promotion. Mineral fertilization improves nutrient availability and promotes changes in the physical and chemical properties of the soil. Pang et al. (2022) verified that long-term fertilization influenced the biomass and number of bacteria, actinobacteria and fungi in the rhizosphere. However, little is known about the influence of mineral fertilization and inoculant concentration on endophytes.

## Objective

The aim of the present study was to verify whether endophytism is influenced by the bacterial inoculant concentration and by the soil fertilization conditions.

## Materials and Methods

### Planting and Inoculation

The soybean plant experiments were carried out in a greenhouse in the city of Jaboticabal, SP (21° 15′ 17″ S and 48° 19′ 20″ W. The greenhouse conditions were maintained at temperature of 24 ± 2C, watering 50 ± 2% RH, and 250 μmol light, 16:8 h L:D. According to the Köppen and Geiger classification, the climate is Aw type, and according to Embrapa (1999), the region has eutrophic red latosol with clayey texture. This soil was used to carry out the experiments in 5 dm^3^ pots.

Inoculants containing *B. subtilis* were cultivated in nutrient broth medium and kept in a BOD chamber at 28°C for 24 h (Lobo et al., 2019). The *B. subtilis* strain used in this study comes from the collection of the Laboratory of Soil Microbiology, UNESP, Campus of Jaboticabal under the GenBank number MZ133755. It was selected for its growth-promoting characteristics, such as phosphorus solubilization, biological nitrogen fixation, and indole acetic acid production (Diaz et al., 2019; Milani et al., 2019). Inoculant concentrations were standardized by spectrophotometer readings at 630 nm (Kloepper et al., 1989), and each pot received 10 ml of inoculant. Fertilization treatments were carried out with 350 kg ha^−1^ of fertilizer formula NPK (0-20-20) at four levels, namely, F0 = 0, F25 = 25% (17.5 g of both N and P per vase), F50 = 50% (35 g of both N and P per vase) and F100 = 100% (70 g of both N and P per vase), according to soil chemical analysis under greenhouse conditions. Inoculant concentrations were adjusted as follows: D0 (control without inoculation), D2 = 1×10^2^, D4 = 1×10^4^, D6 = 1×10^6^, D8 = 1 × 10^8^ and D9 = 1 × 10^10^ CFU mL^−1^. For example, F50D6 indicates a fertilization dose of 50% and inoculant concentration of 1 × 10^6^ CFU mL^−1^.

The seeds were sterilized prior to inoculation. The seeds were washed with tap water and deionized water and dried on absorbent towels. Then, 2 g of seeds was transferred aseptically to a sterile beaker, washed two times with sterile distilled water and sterilized using 0.2 g% HgCl_2_ for 30 s. Then, the seeds were washed six times with distilled water (Ladha et al., 1997). After sterilization, seeds were sown in pots with soil previously chemically analyzed and fertilized according to Raij et al. (1997) for expected yields of 8–10 t ha^−1^. After the emergence of plants, thinning was performed, leaving one plant per pot. Inoculations containing 10 ml of inoculant were injected into the stems of plants with the aid of a volumetric pipette at the V3 stage and were repeated every 10 days for a period of 60 days. The samples were collected after 60 of sowing.

### Collection and Sterilization of Root Sample Surfaces

To collect plants, pots were poured into a sterilized bin, and the soil was loosened from roots using a sterile metal spatula. Subsequently, shoots were separated from roots, and 4 to 6 roots from each plant were placed in a 50 ml conical tube containing 35 ml of phosphate buffer with 0.02% surfactant (Tween 20). The tubes were vortexed for 2 min to separate the root systems from the rhizosphere. Then, with the aid of sterilized tweezers, roots were placed on paper towels and transferred to centrifuge tubes (50 ml).

Superficial sterilization of roots was performed according to Cao et al. (2005), with modifications. Root tissues were maintained in 100% ethanol for 3 min, followed by 2% sodium hypochlorite for 2 min and 70% ethanol for 3 min. Disinfected roots were washed three times with sterile distilled water, and the last wash was inoculated onto nutrient agar plates to validate the effectiveness of the surface sterilization procedure.

### DNA Extraction and Amplicon Sequencing

Sterilized roots were macerated with a sterile mortar and pestle with the aid of liquid nitrogen. The PowerMax Soil DNA Extraction Kit (Mo Bio Laboratories, Carlsbad, CA) was used to extract the genomic DNA according to the manufacturer’s instructions. The concentration of extracted DNA was determined by fluorometry (Qubit^™^ 3.0, Invitrogen), and purity was estimated by calculating the A260/A280 ratio *via* spectrophotometry (NanoDrop^™^ 1000, Thermo Fisher Scientific). The hypervariable region V4 of the 16S rRNA gene was amplified with the primers 515F (5′-GTGCCAGCMGCCGCGGTAA-3′) and 806R (5′-GGACTACHVGGGTWTCTAAT-3′; Caporaso et al., 2010). Three forward primers were used in the amplification. These were modified by adding degenerate nucleotides (Ns) to the 5′ region to increase the diversity of target sequences (de Souza et al., 2016). PCR was performed in 30 cycles using the HotStarTaq Plus Master Mix Kit (Qiagen) under the following conditions: 94°C for 3 min, followed by 28 cycles of 94°C for 30 s, 53°C for 40 s and 72°C for 1 min, and a final elongation step at 72°C for 5 min. PNA clamp sequences (PNA Bio) were added to block amplification of the 16S rRNA gene from ribosomes and mitochondria. Amplification products were analyzed on a 2% agarose gel to determine the amplification success and the relative intensity of bands. Amplicon sequencing was performed on an Illumina MiSeq platform.

### Data Processing

Raw sequence data were verified with sequence quality filters in FastQC software (Andrews, 2010). Sequences longer than 150 bp were removed, and the adapters and primers were removed using Trimmomatic software (version 0.36; Bolger et al., 2014). Sequencing data were processed using the pipeline Quantitative Insights into Microbial Ecology (QIIME) version 1.9.1 (Caporaso et al., 2010). All sequences that passed quality control were grouped into single sequence variants (ASVs – amplicon sequence variants), and a count table was generated. For the taxonomic annotation of ASVs, the DADA2 package (Callahan et al., 2019) and the 16S rRNA gene sequences database SILVA^1^ were used. Representative sequences for each ASV were aligned using MAFFT (Kuraku, 2013), and multiple sequence alignment was used to reconstruct evolutionary relationships with the FastTree 2 tool (Price et al. 2010). The phylogenetic tree, the count table, and the taxonomically annotated ASVs were imported into the R environment for statistical analyses.

### Data Availability Statement

The datasets presented in this study can be found in online repositories: https://www.ncbi.nlm.nih.gov/Traces/study/?acc=PRJNA820178.

### Statistical Analysis Methods

Data were processed and visualized in R 4.1.1 using custom scripts. Specifically, analyses were conducted using the phyloseq package (McMurdie and Holmes, 2013). The normalization of counts was performed by using the DESeq2 package. Then, alpha and beta diversity metrics were calculated. To characterize the alpha diversity, the observed richness and the Chao1, Shannon and Simpson indices were determined. The significance of mineral fertilization and inoculum application under alpha diversity indices was evaluated with ANOVA. *Post hoc* comparisons were conducted by Tukey’s tests of significant differences. The dissimilarity between the samples was determined using the Bray–Curtis, Jaccard, UniFrac and Weighted UniFrac distances. Principal coordinate analysis (PCoA) was performed to visualize the relationships between samples based on beta diversity.

## Results

**Figure 1:**
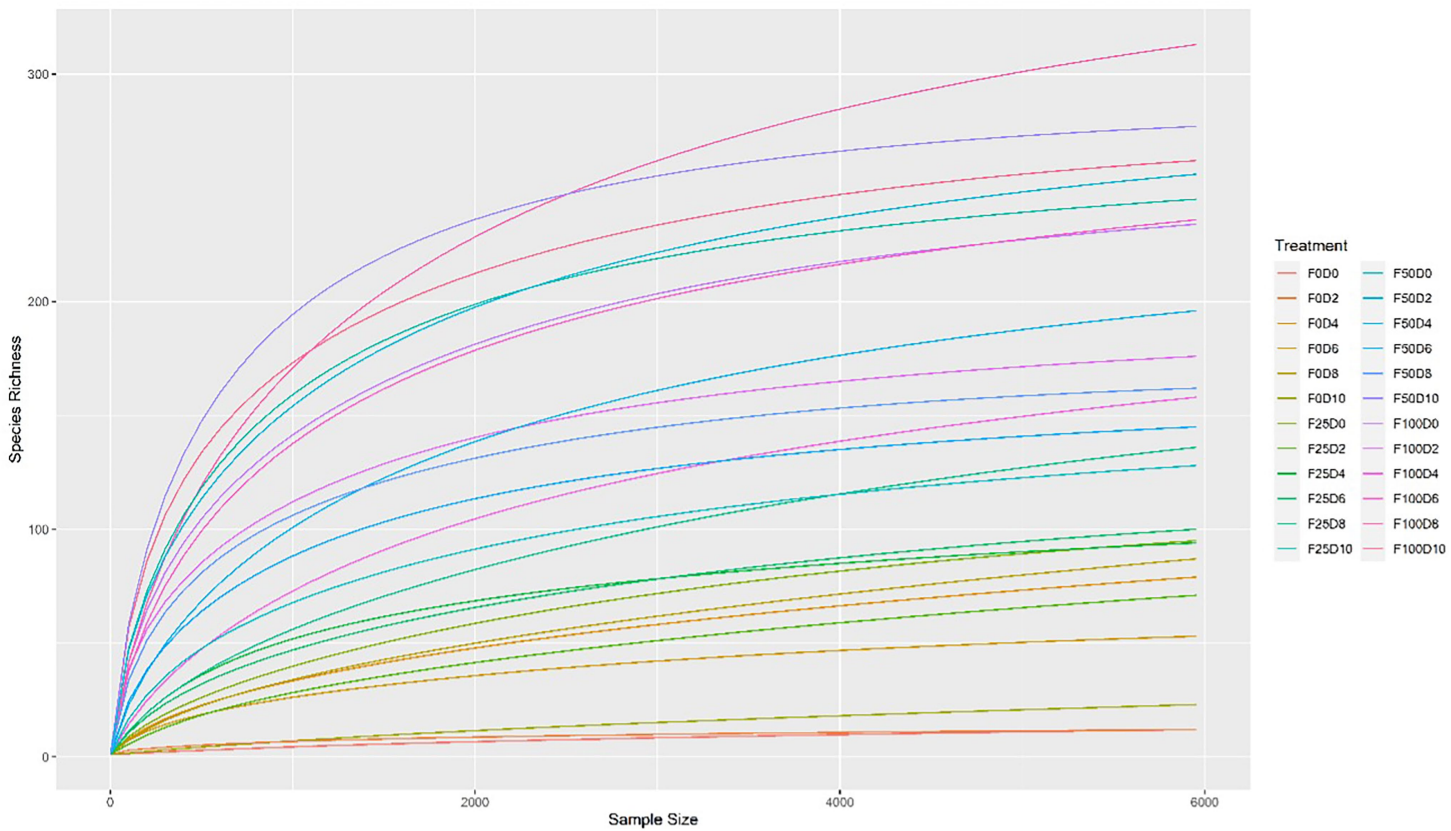
Rarefaction curves of partial sequences of the 16S rRNA gene. Rarefaction analysis of 16S rRNA gene sequence data to estimate the microbial diversity of the 24 treatments.

**Figure 2:**
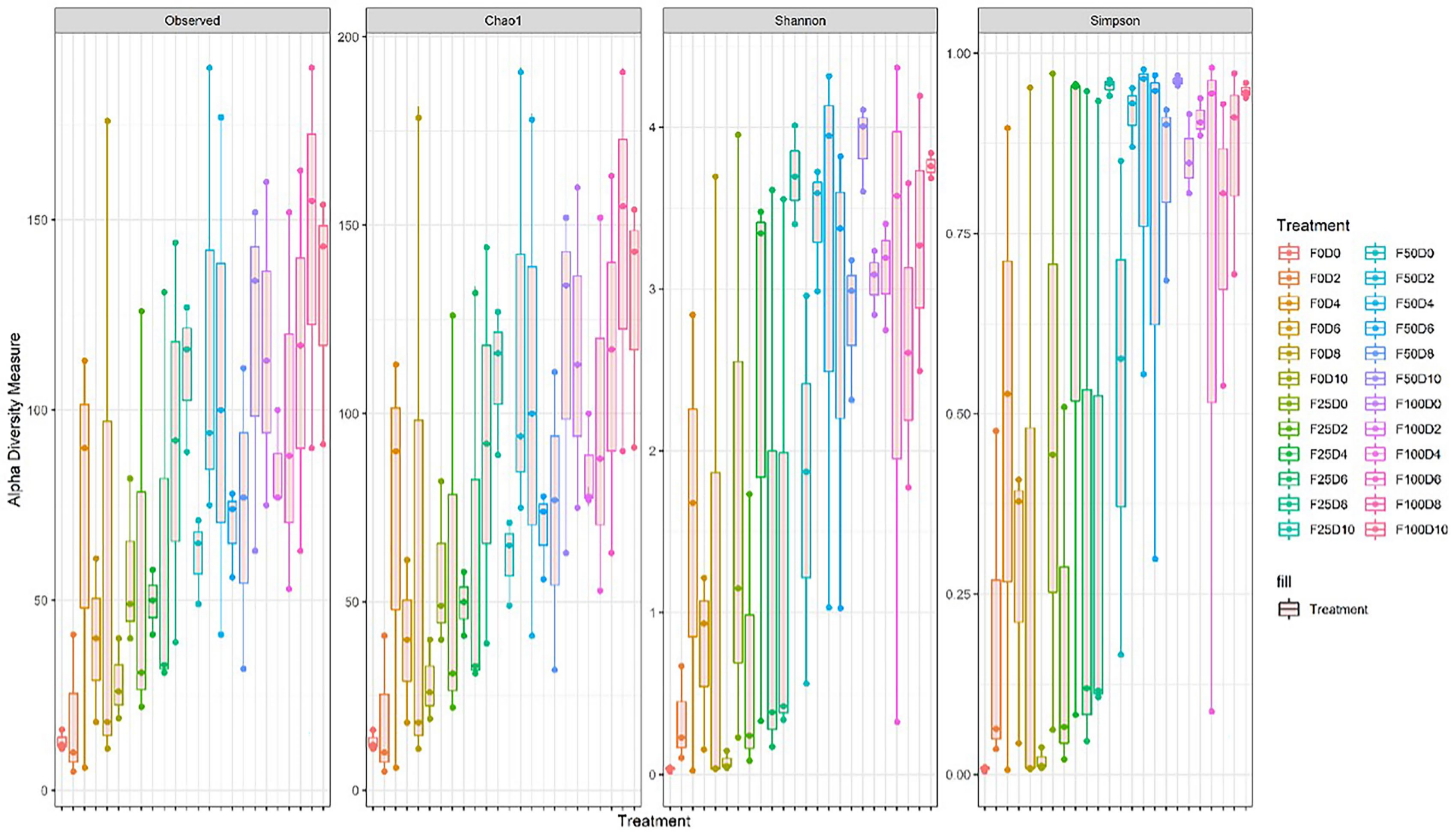
Alpha diversity for all treatments estimated from the observed richness, Chao1 index, Shannon index and Simpson index.

Seventy-two libraries were produced from the samples considering 24 treatments × 3 replicates. High-throughput sequencing generated a total of 8,568,362 crude reads, leaving 2,644,550 reads after quality control, with a total of 1,854 ASVs after excluding unassigned sequences. After data normalization, the rarefaction curve reached a plateau for most treatments, suggesting that taxonomic diversity was mostly recovered (Figure 1). The evaluation of the microbial community structure according to the alpha diversity metrics (observed richness, Chao1 index, Shannon index and Simpson index) showed that microbial diversity of endophytes was higher at fertilization levels of 50 and 100%, with a significant difference (*p* < 0.05) between 0 and 50% and 0 and 100% fertilization (Tables 1, 2; Figure 2). For the inoculum concentrations, no significant difference was observed, and no significant interaction between inoculum concentration and mineral fertilizer level was observed (Table 1).

**Table 1:** Value of *p* for alpha diversity metrics comparing inoculum concentration and mineral fertilization levels: observed richness, Shannon index, Simpson index and Chao1 index.

Indices	Observed richness	Shannon	Simpson	Chao1
Inoculum	0.342	0.195	0.419	0.342
Fertilization	5.19 × 10^−5***^	2.95 × 10^−7***^	2.59e−07^***^	5.44 × 10^−5***^
Inoculum × Fertilization	0.671	0.545	0.367	0.674

Three asterisks ^***^ indicate statistical difference *p* < 0.01.

**Table 2:** Value of *p* for fertilization rates estimated from observed richness, Shannon index, Simpson index and Chao1 index values.

Indices	Observed richness	Shannon	Simpson	Chao1
100–0%	**1.04** × **10**^−5***^	**5.0** × **10**^−7***^	**6.0** × **10**^−7^***	**1.09** × **10**^−5***^
25–0%	0.1080	0.0197	0.0297283	0.1108
50–0%	**3.3** × **10**^−3**^	**2.3** × **10**^−6***^	**2.1** × **10**^−6***^	**3.5** × **10**^−3***^
25–100%	0.0232	0.0198265	0.0143	0.0232
50–100%	0.3638	0.9833	0.9908	0.3639
50–25%	0.5714	0.0509	0.0321	0.5716

Three asterisks ^***^ indicate statistical difference *p* < 0.01 and two asterisks ^**^ indicate statistical difference *p* < 0.05. The bold values mean statistical significance.

**Figure 3:**
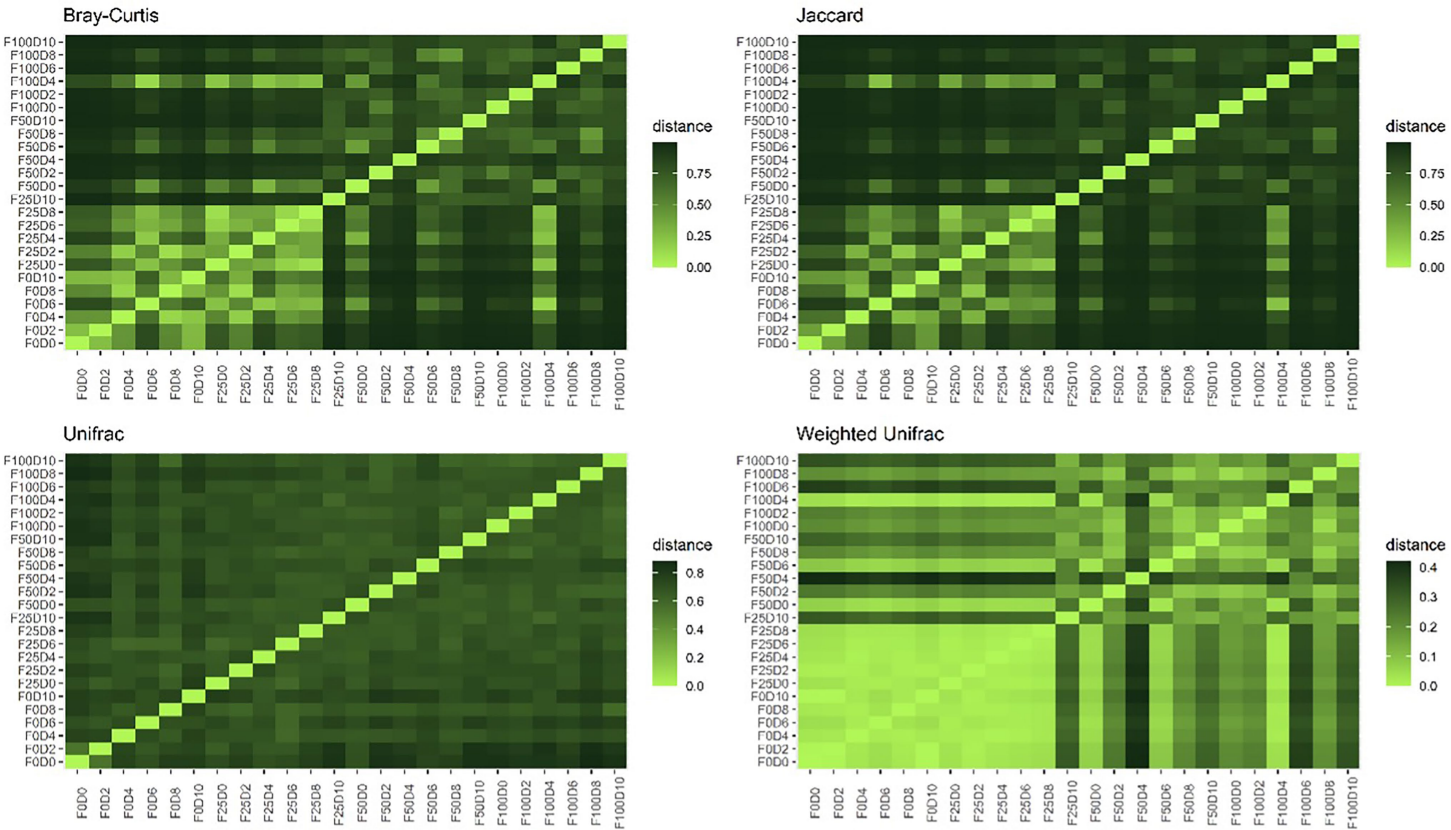
Proportion of dissimilarity – beta diversity based on Bray–Curtis, Jaccard, UniFrac and Weighted UniFrac metric distances for the 24 treatments.

Dissimilarity between bacterial communities was determined based on the number of shared taxa (Jaccard and Bray–Curtis) and the phylogenetic relationship between them (UniFrac and Weighted UniFrac). The distribution of abundance values was also considered by calculating the Bray–Curtis and Weighted UniFrac distances (Figure 3). The compositional dissimilarity was greater when comparing communities treated with low (0 and 25%) versus high (50 and 100%) concentrations of mineral fertilizer. This difference was not detected when phylogenetic distances between taxa were considered. However, no pattern of dissimilarity was found between communities treated with different inoculum concentrations.

**Figure 4:**
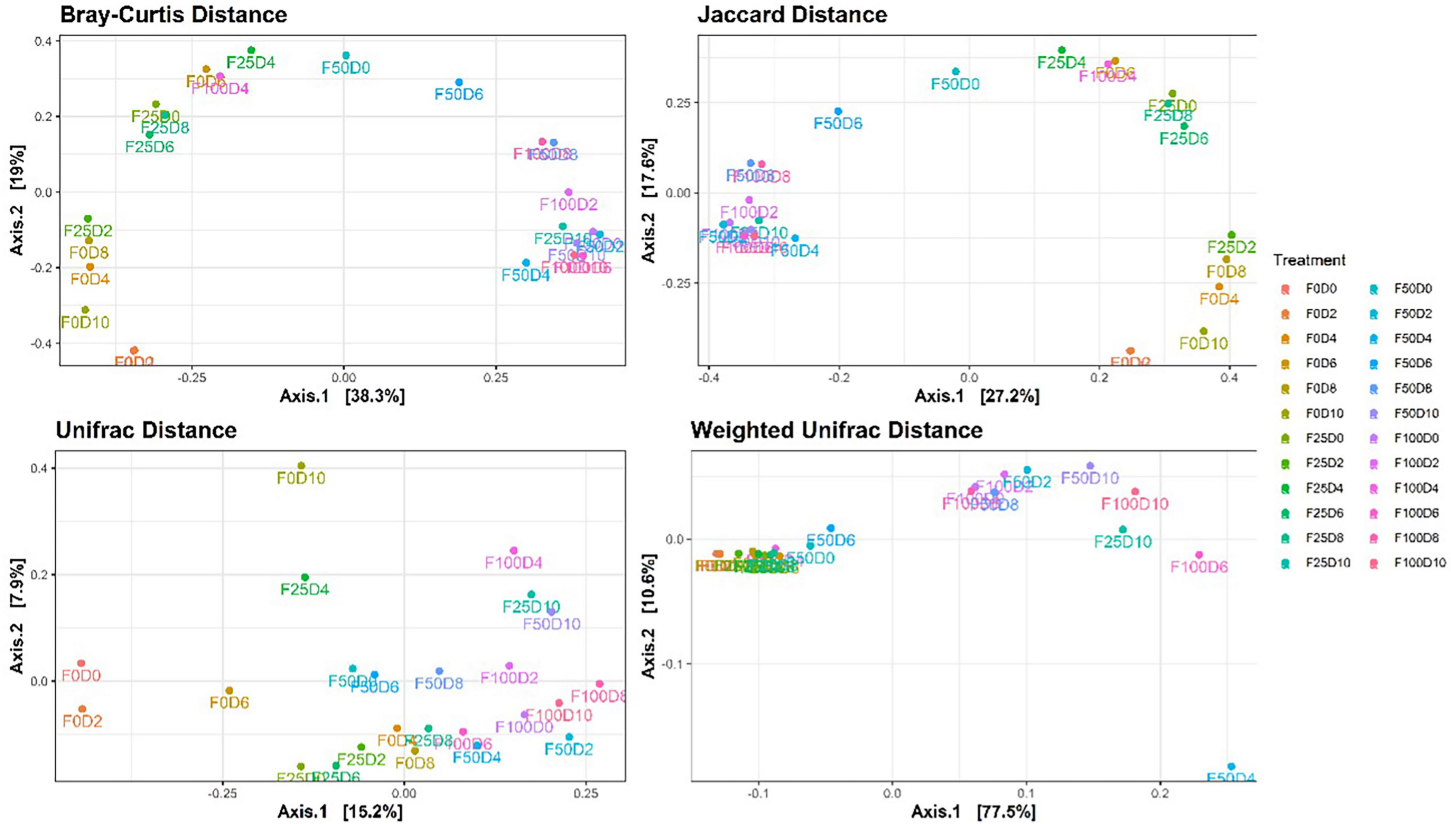
Principal coordinate analysis (PCoA) based on Bray–Curtis, Jaccard, UniFrac and Weighted UniFrac metric distances for the 24 treatments.

Ordination analysis using Weighted UniFrac distances allowed us to recover more than 85% of the variation between communities. In this analysis, axis 1 (77.5%) represented most of the variation and showed high proximity between communities treated with low fertilization concentrations (0 and 25%). Additionally, most samples exposed to 50 and 100% fertilization were also clustered together. This ordination pattern was similarly observed under PCoA using Bray–Curtis and Jaccard distances. It was not possible to identify a pattern of relationships between communities with only phylogenetic distances (UniFrac; Figure 4). With respect to the composition of bacterial communities, the classes with higher than 1% abundance included *Actinobacteria, Alphaproteobacteria, Bacilli, Bacteroidia, Chlamydiae, Gammaproteobacteria, Polyangia, Saccharimonadia*, and *Verrucomicrobiae*.

**Figure 5:**
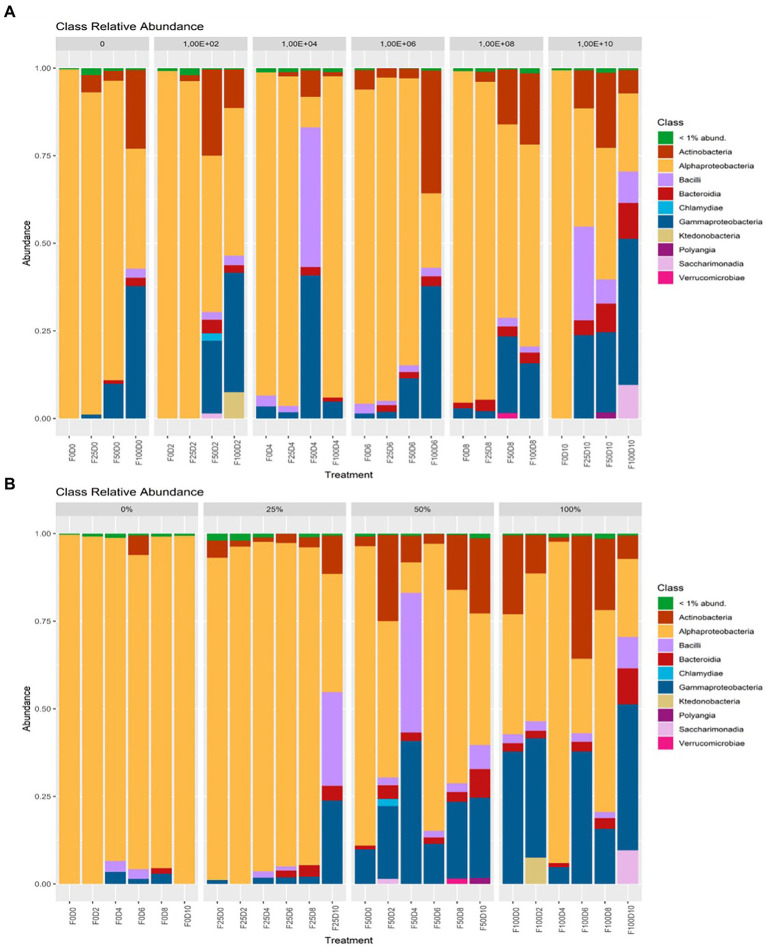
Taxonomic affiliation at the phylum level regarding inoculation **(A)** and fertilization **(B)**.

**Figure 6:**
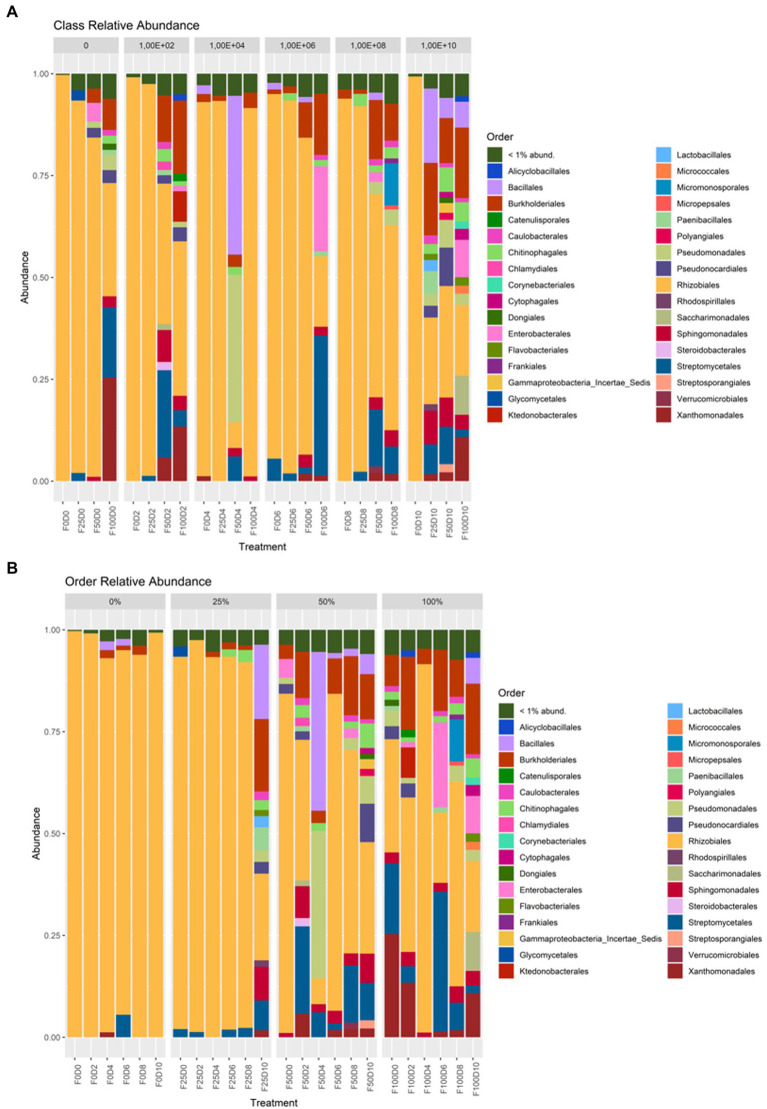
Taxonomic affiliation at the order level regarding inoculation **(A)** and fertilization **(B)**.

Considering the treatments and groupings of ASVs at the phylum level, at fertilization rates of 0 and 25%, the most abundant taxa belonged to the phylum Alphaproteobacteria. At fertilization levels of 50 and 100%, the phyla Gammaproteobacteria and Actinobacteria showed the greatest abundance (Figure 5). At the order level, under fertilization rates of 0 and 25%, it was observed that Rhizobiales contained the most taxa with greater abundance. At fertilization rates of 50 and 100%, other orders, such as Bacillales, Burkholderiales, Streptomycetales and Xanthomonadales, had higher abundance (Figure 6).

**Figure 7:**
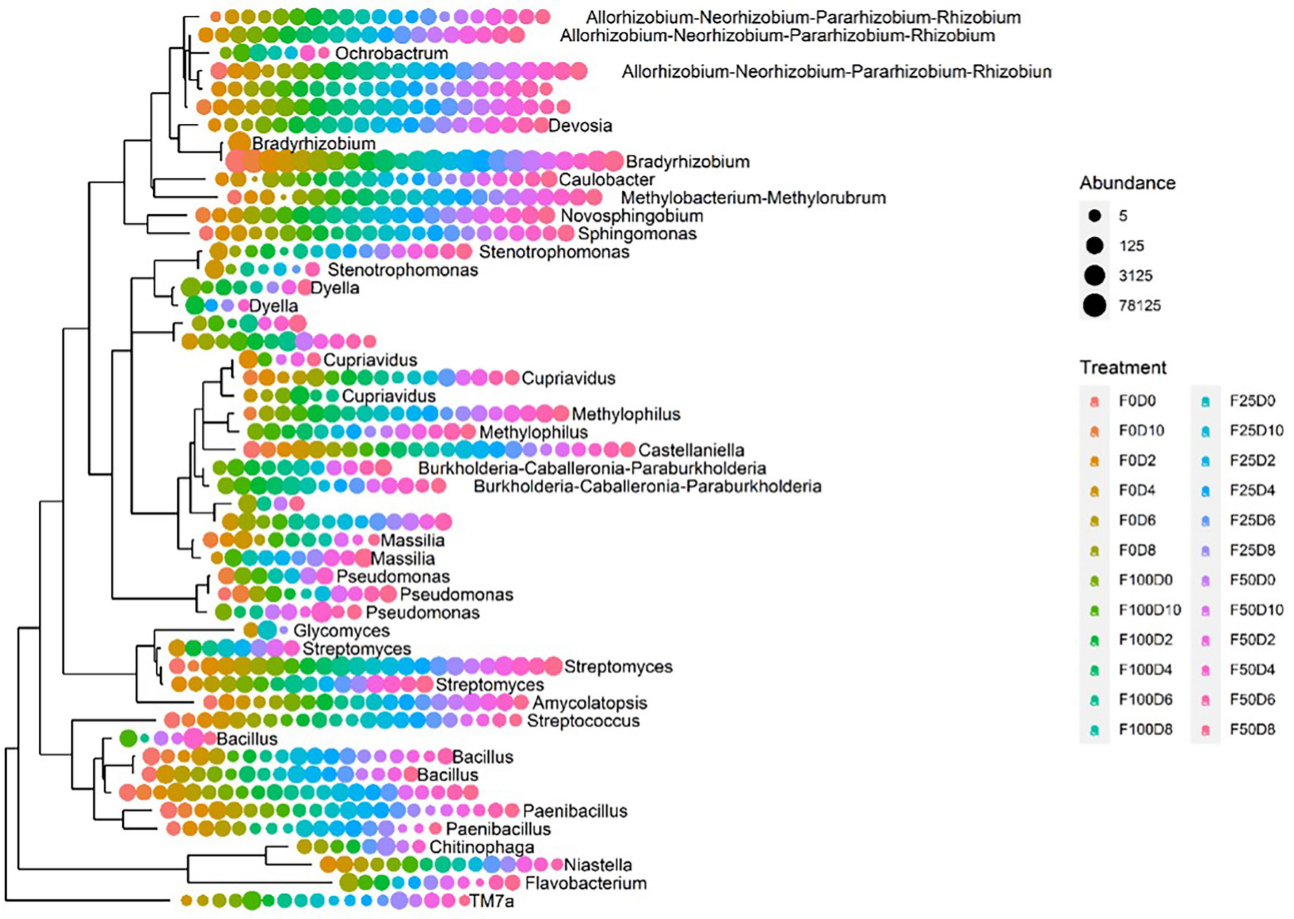
Phylogenetic tree of the 50 most abundant ASVs for all treatments at the genus level.

**Figure 8:**
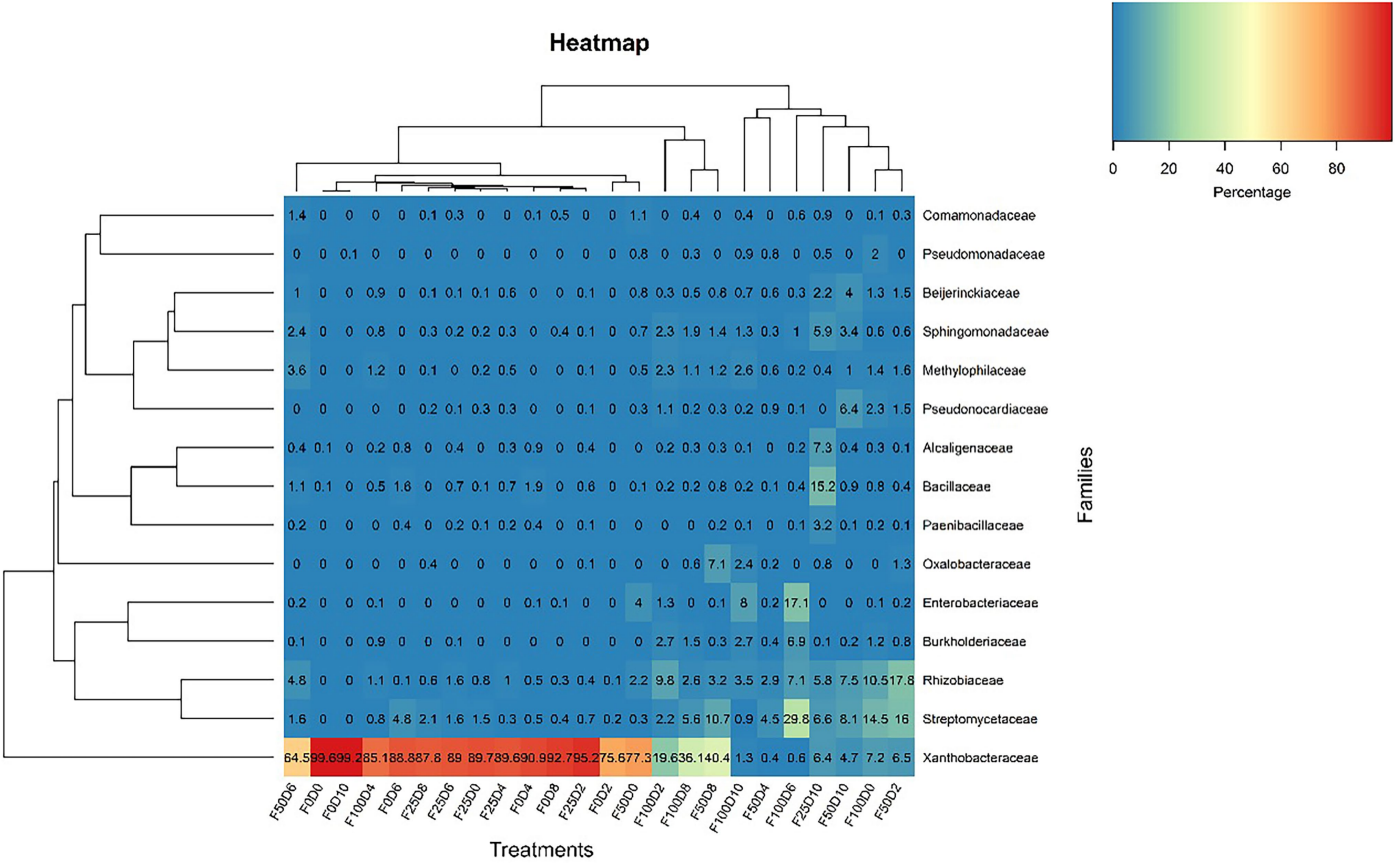
Heatmap clustering with the most abundant ASVs at the family level considering all 24 treatments.

Figure 7 shows the evolutionary relationships among the 50 dominant genera. It was not possible to perform taxonomical attribution at the genus level in taxa without labels. The majority of genera were uniformly found across all treatments. Nonetheless, *Burkholderia, Methylophilus, Dyella* and *Ochrobactrum* were present exclusively in samples treated with fertilizer (25, 50 and 100%). The abundance of the *Bacillus* and *Paenibacillus* genera was favored in samples treated with the lowest fertilizer concentrations (0 and 25%). Conversely, *Pseudomonas* was commonly present in samples treated with high fertilizer concentrations. Despite being bacterial groups that are commonly associated with plants, *Pseudomonas* and *Stenotrophomonas* occurred in only a few samples. The heatmap plot at the family level (Figure 8) was based on the 16 most abundant bacterial families. This analysis allowed us to determine which taxonomic groups were most abundant in each treatment. Across all F25 fertilization treatments, the highest abundance was found for the *Xanthobacteraceae* family; this was also the case for the F0D2 and F50D0 treatments.

## Discussion

Inoculation with microorganisms allows exogenous microorganisms, which are not part of the native microbiota, to colonize the rhizosphere. This colonization allows the reorganization of the temporal composition and the rhizosphere structure (Ambrosini et al., 2016; Adeleke et al., 2021). Interestingly, *B. subtilis* was inoculated in large amounts and at concentrations of up to 1 × 10^10^ CFU mL^−1^, yet the microbial diversity of the roots was not affected (*p* > 0.05; Table 1). Interactions between plants and microorganisms vary according to the genotypes of the plant and microorganism and depend on the proximity between roots and the soil of the rhizosphere. In addition, microorganisms have the ability to improve plant growth, increase the availability of nutrients and the production of phytohormones, alleviate stress and improve defense against phytopathogenic microorganisms (Bulgarelli et al., 2013; Borruso et al., 2018; Compant et al., 2019).

Interestingly, the *B. subtilis* bacterium used in the present study has several plant growth-promoting characteristics, such as biological nitrogen fixation, phosphorus solubilization, and indole acetic acid production, as has been demonstrated in previous studies in soybean, corn and cotton crops (Lobo et al., 2019; dos Santos et al., 2021; Escobar Diaz et al., 2021a). Different plant organs have different ways of being colonized by microorganisms (Compant et al., 2021). Free-living soil microorganisms can colonize roots (Bulgarelli et al., 2012; Edwards et al., 2015). The successful colonization of plant roots by microorganisms *via* an inoculant is complex, and many unknown factors are involved (Mengistu, 2020).

The results have shown that the fertilizer may have affected the bacterial diversity and inoculum did not in the root. The mechanisms underpinning variation in microbial community structure and function in the endosphere remain unclear, and some studies bring controversial results. The bacterial community was first influenced by soil conditions, including fertility and plant genotype. The minor factor was bacterial inoculation (Fan et al., 2018). Moreover, the bacterial colonization in the plant interior is attractive since the host nutrients can be used efficiently and without competition from high bacterial numbers colonizing outside the roots (Liu et al., 2017; Morales-Cedeño et al., 2021). The inoculation of *Serratia* sp. and *Arthrobacter* sp. in Indian mustard affected the specific composition and diversity of endophytic bacterial communities in roots with no significant effect on rhizospheric communities (Wang et al., 2020). Another study verified that inoculation with *Mesorhizobium loti* in *Robinia pseudoacacia* did not affect the microbial diversity on the root (Fan et al., 2018).

Most likely, *B. subtilis* inoculation promoted some temporary modifications to the root microbial diversity. These changes, if they occurred, were not permanent, probably due to the plant not allowing the permanent colonization of this bacterium (Del Carmen Orozco-Mosqueda and Santoyo, 2021). The plant likely did not need the growth-promoting abilities of *B. subtilis*. Trabelsi and Mhamdi (2013) reported little or no effect of inoculation of a single isolate on the resident microbiome of plants. Changes in microbial composition may be undesirable if important native species are lost, thus affecting subsequent cultures. However, changes in microbial community structures caused by inoculation may be buffered by ecosystem resilience, which is driven by the level of diversity and plant – soil – microbiota system interactions (Liu et al., 2020; Tosi et al., 2020). Unlike *B. subtilis* inoculation, mineral fertilization increased the microbial diversity of soybean plant roots (*p* < 0.05; Table 1).

Fertilization is an agricultural practice essential for increasing crop quality and productivity and works by improving nutrient availability and promoting changes in soil physical and chemical properties and microbial communities (Li et al., 2017; Sasse et al., 2018; Liu et al., 2020). Although mineral fertilization provides several benefits, large amounts of fertilizers can cause several environmental problems, such as soil degradation, nitrogen leaching, the release of greenhouse gases and, consequently, decreased productivity (Horrigan et al., 2002; Wang et al., 2017). Wang et al. (2017) verified the effect of organic and inorganic fertilization on the soil microbial diversity during 10 years of fertilizer applications. The results of this study show that inorganic fertilization decreased the richness of bacteria and increased the richness of fungi. The application of mineral fertilizers increased the abundance of some oligotrophic bacteria, such as Bacteroidetes and Acidobacteria, and the application of organic fertilizers increased the abundance of coprotrophic bacteria, such as those in the phylum Proteobacteria (Wang et al., 2017). In the present study, mineral fertilization promoted changes in the abundance of most taxa involved in the root microbiome, with shifts from Alphaproteobacteria to Gammaproteobacteria and Actinobacteria.

Many studies have reported the effects of soil fertilization on soil properties and crop yields. Pernes-Debuyser and Tessier (2004) reported that the application of organic manure could maintain soil organic matter, whereas ammoniacal fertilizers can strongly decrease soil pH and its cation exchange capacity. Belay et al. (2002) reported that the amount of total organic carbon was reduced by prolonged mineral fertilization, while crop yields increased significantly. Studies have shown that changes in the soil microbiome occur after the application of mineral fertilizers (Zhong and Cai, 2007). However, few studies have shown changes in plant root microbiome diversity. Most likely, the factors and parameters used by plants to modulate the root microbiome or the population of endophytes differ from the factors that modulate the microbial populations of the rhizosphere (Bulgarelli et al., 2013; Hardoim et al., 2015).

Changes in the diversity of endophytic communities can be easily observed and compared. The lowest rates of mineral fertilization increased the dominance of Alphaproteobacteria, which is a heterogeneous class that includes free-living, symbiotic, gram-positive bacteria and some integral intracellular bacteria that generally have important metabolic capabilities, such as biological nitrogen fixation and carbon fixation (Hallez et al., 2017). However, the highest rates of mineral fertilization increased the abundance of Gammaproteobacteria, which contains a wide range of bacterial genera, including many with growth-promoting abilities, such as *Stenotrophomonas* and *Pseudomonas*, which also have the ability to produce phytohormones (Taghavi et al., 2009). However, analyses of alpha or beta diversity are not enough to explain the role of interactions between microbial species that coexist in plants (Barberán et al., 2012). The present study shows an initial increase in diversity promoted by higher rates of mineral fertilization under potted conditions. However, further studies are needed to verify the benefits to plants as a result of these changes.

Studies have shown the benefits of endophytic bacteria compared to rhizospheric bacteria regarding increased relationships and interactions between microorganisms and the host plant (Brader et al., 2017; Adeleke et al., 2021). Other studies have also shown that microbial diversity promotes several gains related to plant growth promotion and health (Eid et al., 2019; Morelli et al., 2020; Adeleke et al., 2021). In this sense, mineral fertilization not only supplies nutrients to plants but also improves plant health. However, some studies have shown that this effect is transient and that mineral fertilization in large amounts and with prolonged effects would do more harm than good (Wang et al., 2017).

## Conclusion

The results show that *B. subtilis* inoculations did not affect the endophytic community of roots; however, the evaluation of the microbial community structure according to the alpha diversity metrics observed richness, Chao1 index, Shannon index and Simpson index showed that microbial diversity of endophytes was higher at fertilization levels of 50 and 100%, with a significant difference (*p* < 0.05) between 0 and 50% and 0 and 100% fertilization.

## Data Availability Statement

The datasets presented in this study can be found in online repositories: https://www.ncbi.nlm.nih.gov/Traces/study/?acc=PRJNA820178.

## Author Contributions

All authors listed have made a substantial, direct, and intellectual contribution to the work and approved it for publication.

## Conflict of Interest

The authors declare that the research was conducted in the absence of any commercial or financial relationships that could be construed as a potential conflict of interest.

## Publisher’s Note

All claims expressed in this article are solely those of the authors and do not necessarily represent those of their affiliated organizations, or those of the publisher, the editors and the reviewers. Any product that may be evaluated in this article, or claim that may be made by its manufacturer, is not guaranteed or endorsed by the publisher.

## Footnotes

^1^ https://www.arb-silva.de/no_cache/download/archive/release_138
